# The Half-Yearly Abstract of the Medical Sciences

**Published:** 1845-11

**Authors:** 


Art. XIII.—*The Half-Yearly Abstract of the Medical Sciences:* being a practical and analytical digest of the contents of the principal British and Continental medical works published in the preceding six months. Together with a series of Critical Reports on the Progress of Medicine and the Collateral Sciences during the same period. Edited by W. H. Ranking, M.D., Cantab., Physician to the Suffolk General Hospital. N. York: J. & H. G. Langley. 1845. 8vo. pp. 372.

The *Half-Yearly Abstract* is to be the rival of Braithwaite's excellent *Retrospect*, and, from the specimen afforded in the first number, a very formidable rival. We do not propose instituting a comparison between them, but, as we have hitherto recommended the Retrospect to our readers, not only in direct terms, but quite as substantially by quoting largely from its pages, so our present aim is to bring the Abstract favorably before our readers; and we have no hesitation in saying that it is a judicious and rich summary of medical science. We received it too late to make any selections from it for our present number.

